# Scaling up SoccerNet with multi-view spatial localization and re-identification

**DOI:** 10.1038/s41597-022-01469-1

**Published:** 2022-06-21

**Authors:** Anthony Cioppa, Adrien Deliège, Silvio Giancola, Bernard Ghanem, Marc Van Droogenbroeck

**Affiliations:** 1 University of Liège, Montefiore Institute, Quartier Polytech 1, Allée de la découverte 1, 4000 Liège, Belgium; 2 King Abdullah University of Science and Technology, Image and Video Understanding Laboratory, 23955 Thuwal, Saudi Arabia

**Keywords:** Databases, Electrical and electronic engineering, Computer science, Scientific data

## Abstract

Soccer videos are a rich playground for computer vision, involving many elements, such as players, lines, and specific objects. Hence, to capture the richness of this sport and allow for fine automated analyses, we release SoccerNet-v3, a major extension of the SoccerNet dataset, providing a wide variety of spatial annotations and cross-view correspondences. SoccerNet’s broadcast videos contain replays of important actions, allowing us to retrieve a same action from different viewpoints. We annotate those live and replay action frames showing same moments with exhaustive local information. Specifically, we label lines, goal parts, players, referees, teams, salient objects, jersey numbers, and we establish player correspondences between the views. This yields 1,324,732 annotations on 33,986 soccer images, making SoccerNet-v3 the largest dataset for multi-view soccer analysis. Derived tasks may benefit from these annotations, like camera calibration, player localization, team discrimination and multi-view re-identification, which can further sustain practical applications in augmented reality and soccer analytics. Finally, we provide Python codes to easily download our data and access our annotations.

## Abstract (summary)

**Table:** 

Measurement(s)	Localization of soccer features
Technology Type(s)	Manual annotations

## Background & Summary

Sports has a unifying aspect in our society, gathering millions of fans together to support their favourite athletes and teams. In particular, soccer is one of the most popular and lucrative sports in the world, generating billions of dollars of revenue each year^1^. For that reason, broadcasting companies need to stay competitive by constantly improving the viewer experience through innovative visualization tools, while coaches have to perform advanced analyses to boost their team’s performance and climb at the top of the ranking. Those tasks can benefit from the ever-expanding set of games broadcast each week. However, the amount of data collected per match day is too large to be handled manually. Thus, automatic methods are required to enable a fast and comprehensive processing of sports videos^2,3^. Nowadays, deep learning-based methods are used in a wide variety of soccer-related computer vision tasks, such as action spotting^4–10^, player segmentation^11^, counting^12^ and tracking^13,14^, ball tracking^15^, tactics analysis^16^, pass feasibility^17^, talent scouting^18^, game phase analysis^19^, or highlights generation^20,21^. To achieve such objectives, the methods developed often rely on either generic learning-based algorithms^22,23^ trained on public datasets^24,25^, and/or on fine-tuned algorithms trained on private task-specific soccer datasets^7,9,19^. In the first case, the performances achieved are suboptimal, while in the second case, they cannot be reproduced by a third party. Finally, some public datasets are simply too small, preventing good generalization to many games^26,27^. Therefore, to further develop the soccer research community and establish strong reproducible benchmarks, *large public soccer-centered datasets* are needed.

**Fig. 1:**
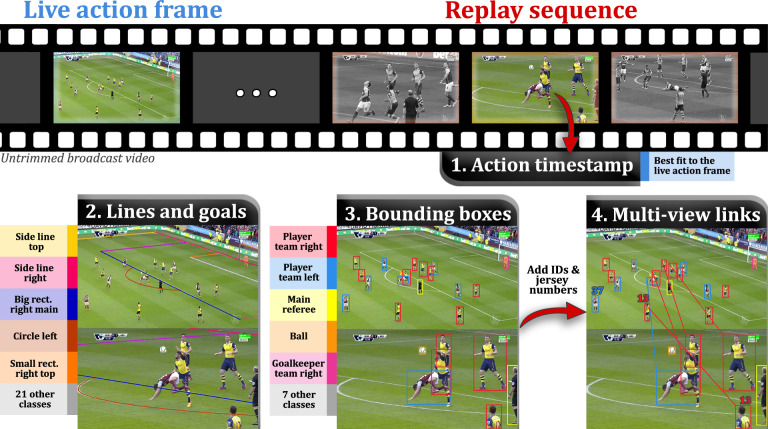
SoccerNet-v3 annotations. In each replay sequence of an action within 400 of SoccerNet’s full broadcast games, we select the replay frame showing the live action frame (top). Then, on all those replay and live action frames, we annotate lines and goal parts (left), classified into respectively 20 and 6 classes, illustrated in different colors. We further enclose each human on the field within a bounding box, we specify players team, and annotate salient objects (middle). Finally, we establish multi-view player correspondences across replay and live action frames of the same scene, using jersey numbers whenever possible (right). SoccerNet-v3 thus provides a rich environment to explore soccer-related computer vision tasks.

We build SoccerNet-v3 upon the corpus of annotations provided in SoccerNet-v2^5^ on the initial SoccerNet^28^ dataset. Essentially, SoccerNet is a video dataset of 500 untrimmed broadcast soccer games played between 2014 and 2017 in the main professional European leagues. It is equipped with a first corpus of annotations, consisting of temporal anchors (timestamps), that localize in time the occurrence of three action classes (goals, cards, substitutions), and which defines a first yet basic task of action spotting. SoccerNet-v2 extends that corpus to 17 action classes and provides annotations to tackle the tasks of camera view classification and clip temporal boundary detection. In particular, each replay clip of an action is precisely delimited and further linked to the live action frame, and used to set up a replay grounding task. Hence, albeit being enriched with various soccer-related information, current annotations on the SoccerNet videos are only anchored within the temporal domain. This limiting factor narrows down the amount of automatic analyses of the game that can benefit from such a large dataset. Therefore, to facilitate the development of a wider variety of tasks and to allow for more fine-grained analyses of soccer scenes, we release a substantial amount of supplementary annotations. Most of them are anchored spatially and instinctively define specific tasks, proposed hereafter. We provide several types of annotations, illustrated in Fig. 1, which we summarize as follows:

1. **Action timestamps within replays**. For each replay clip of an action and its linked action frame of the live game (both from SoccerNet-v2), we select the replay frame that shows the same moment as the one seen in the live action frame. This yields 21 k replay frames, linked to 12 k unique action frames (some replays refer to the same action). Those action and replay frames simulate a synchronous multi-camera capture system, for which no soccer dataset exists. Hence they allow to explore tasks benefiting from synchronous images, such as multi-view re-identification (see hereafter). The following annotations are performed on these frames of interest.
2. **Lines and goals**. We annotate each soccer field line (straight or curved) by placing on it as many points as needed to fit it with the segments formed by those points, and we tag it according to a 20-class ontology. We annotate each goal part (left/right post, crossbar) in the same way, specifying whether each part belongs to the left or right-hand side goal on the field. All these annotations are particularly useful for a camera calibration task to further localize the players on the field. Note that annotating goal parts is new. It is relevant because goals have highly accurate dimensions and are located outside the 2D plane of the field, allowing for a camera calibration that potentially expands to the real 3D space instead of a 2D homography.
3. **Bounding boxes for humans (with team ID) and objects**. We annotate every human located on the soccer field with an individual bounding box, further tagged as left/right player/goalkeeper, main/side referee, or staff member. The left/right player feature can be used in a team discrimination task. Player bounding boxes may serve as setup for a player localization task. Whenever visible, we also annotate the ball, flags waved by side referees, yellow/red cards raised by the main referee, and walls of players in case of direct free kick, as they play an important role in soccer video understanding.
4. **Multi-view player correspondences and jersey numbers**. Given an action frame and its set of replay frames, we establish all possible player correspondences, by providing them unique IDs. Whenever visible, those IDs are their jersey number. This forms the basis of a challenging re-identification task across multiple and potentially very different views of the same scene, as well as a wide jersey number recognition task.

Beyond extending previous SoccerNet versions, our SoccerNet-v3 annotations are complementary to those provided in the literature. In particular, Pappalardo *et al*.^29^ released a large-scale spatio-temporal dataset of soccer events^30^. However, they focus on statistical analyses of players and teams rather than soccer video understanding, as they do not release any video. SoccerNet-v3 also provides more diverse and sharper annotations than Yu *et al*.^31^, who released annotations of actions for 222 halves of soccer games, along with shot transitions, and player bounding boxes, but miss information needed for thorough analyses, such as identifiers, lines, goals, and ball information. The same remark also applies to SoccerDB^32^, which added only 7 action classes along player bounding boxes for half of SoccerNet’s videos and 76 extra games. Biermann *et al*. defined a complete taxonomy of soccer events^27^ but they annotated only 5 games. Automatic tools^33,34^ are sometimes used to generate estimates of e.g. bounding boxes and line segmentation masks^35^, but no human quality assessment is made. In our case, we have always at least two humans in the loop that have either performed or checked each annotation. Finally, physics-based 3D simulators^36^ or game engines^37^ can help collect numerous action-related information, such as the SoccER dataset^38^. Contrary to our case, methods developed on these datasets might not be transferable to real-world videos due to domain gaps, as they may lack photorealistic images or plausible game phases.

## Methods

### Workforce

We recruited 83 students of the University of Liège (Belgium) to carry out our annotation campaign. The annotations took place between July and September 2021, for a total of approximately 6,000 hours. In order to respect Covid-19 safety measures, the students worked from home. Following fairness and ethics policies, they were paid as for a regular student job, i.e. we spent on average 11.54 € per student per hour (this might slightly vary with their age), leaving them with roughly 10.58 € net per worked hour.

From an organizational perspective, we appointed two students as supervisors, two as proofreaders, and the remaining as annotators. The annotators were in charge of annotating the data and reporting hard cases, mismatches, potential problems, that would require further examination. This was performed by the proofreaders, which were also in charge of randomly checking the quality of the annotations, correcting them whenever appropriate, and notifying the supervisors in case of repeated issues. The supervisors were mainly in charge of organizing the work of the annotators and bringing them administrative and technical support. Should the supervisors or the proofreaders need assistance, they could directly contact us. The annotations were performed on SuperAnnotate (see https://superannotate.com/), a professional platform specialized in data annotation.

### Specific annotation instructions

#### Action timestamps within replays

We use SoccerNet-v2 annotations to extract replay clips and their linked action frames from the SoccerNet videos. When uploaded on SuperAnnotate, the clips are converted by the platform to sequences of frames, subsampled at 5 frames per second. This frame rate is a convenient trade-off between temporal granularity, memory load, and annotation time. We create a dedicated folder for each clip and associated action frame. Each annotator receives a list of folders to process. We ask the annotator to look at the action frame first, then navigate among the frames extracted from the replay clip and find the one that best shows the same moment. Only one frame per replay clip is selected. Given the subsampling operated by SuperAnnotate, the replay frame selected by the annotator may be shifted temporally by at most 100 milliseconds with respect to the action frame to retrieve, which is negligible. Besides, SoccerNet-v2 contains annotations for “unshown” actions. Those actions occur during the game but cannot be seen in the broadcast video because of the editing (e.g. we know that the goalkeeper makes a clearance, but at that moment, a previous goal attempt is shown). In such cases, action frames do not actually show the action (e.g. the clearance). Thus, given a replay clip and the annotated frame of that action, it is impossible to retrieve that action frame within the replay. In our annotation pipeline, when this situation arises, we ask the annotators to remove such actions, in order to keep only truly corresponding live action and replay frames.

#### Lines and goals

After the first annotation phase, we extract all the replay frames and keep a unique copy of their associated action frames. We place all these frames in a shared folder and we assign them randomly to the annotators, which thus receive images that most probably differ from those they processed previously. In this step, we annotate lines and goals on the frames of interest, which are static semantic elements of the scene.

**Fig. 2:**
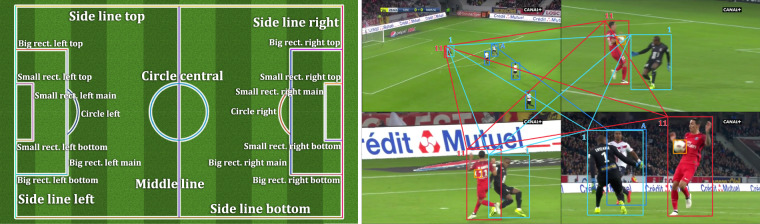
Detailed lines ontology and player correspondences. Left: We define 20 line classes, covering each line of the field with a specific name. We assume that the main camera is located at the bottom of the figure, close to the middle line. Line annotations can be leveraged for a camera calibration task, or can help localize the actions on the field. Right: We annotate player correspondences between the action frame (top left) and the replay frames of the same action from different cameras. When a jersey number is visible, we propagate it to the other instances of that player. Otherwise, we use a generic literal name. We do not tag players without correspondences.

Regarding the lines, we use a 20-class ontology, which is defined with respect to the main camera view of the field (the most seen on TV). This is illustrated in Fig. 2 (left). The 4 external lines are named side lines and can be either left, right, top, bottom. The 12 lines inside the field forming the rectangles can relate to either small or big rectangles, coupled with either a top, bottom, main characterization, and with a left or right-hand side positional information. The remaining lines are the middle line that crosses the field, the central circle and the left or right half circles.

In practice, our line annotations are sets of points placed on each visible line by the annotators in a point-and-click fashion. The annotators focus on one line at a time, place the necessary points on it, then classify them altogether in the appropriate line class. A point placed on a line cannot be re-used in another line; instead, a new point must be placed close by if needed. Lines that appear straight on the frames are annotated with just two points placed on both ends of the line segment captured by the camera, where annotators estimate the position of ends occluded by people or objects on the field. Circles and lines appearing curved due to the camera lens distortion are annotated with as many points as needed so that the successive segments drawn between the points fits the circle or line as accurately as possible. To help the annotators, those segments are traced progressively by the annotation tool after each new point placed. Such segments serve only to ease the annotation process visually. They are not saved but can be retrieved effortlessly, although more appropriate interpolation methods can be used to obtain smoother post-processing visualizations, as in Fig. 1. Encoding lines as sets of points provides a compact abstract representation of the field. Whenever necessary, such a representation can also be transformed into a segmentation mask of the field lines.

Goals are also important elements that help locate the action of the game, when they are visible. In such cases, we annotate them in a similar way as for lines, i.e. with at least two points placed on each goal part (among left post, crossbar, right post), further specifying whether they belong to the left or right goal with respect to the main camera view. This gives a total of 6 classes for goal annotations. The left and right posts of a goal are determined as if the annotator is in front of the goal, as for a penalty shot. If a line or goal part is visible but not identifiable reliably, the annotators just mark it as “line” or “goal”.

#### Bounding boxes for salient humans (with team ID) and objects

In the next step, we run SuperAnnotate’s smart prediction algorithm, which is trained on the COCO dataset^25^ to perform an automatic human detection and to initialize bounding boxes annotations. Then, we randomly assign the frames to the annotators, which thus receive again different images than those processed previously.

We ask the annotators to manually classify the pre-computed bounding boxes, by further specifying which of them belong to left/right field players, left/right goalkeepers, main/side referees, staff. Regarding the staff, we keep coaches and reserve players under that label, but stewards, cameramen, photographs, public, etc., and any other bounding box is removed. Annotators must also add bounding boxes around persons of interest but that are missed by the algorithm. They must take care that each box contains exactly one person. They keep a person of interest cut at the border of the frame only when they can identify him. For instance, half a player can be recognized as such, but just an arm or a leg cannot. The team ID (i.e. left/right feature) of players and goalkeepers is determined by the position of their goal to defend with respect to the main camera view of the game. The annotators have the possibility to browse through other frames of the same game to alleviate their doubts on some difficult views.

Regarding extra bounding boxes for salient objects, we ask the annotators to draw a tight box around the ball, flags waved by side referees, yellow/red cards raised by the main referee, and walls of players when a direct free kick occurs. We annotate only the ball in play, not those that might be visible at the border of the field. We annotate flags and cards only when they are used by a referee, not when they are visible incidentally. If a wall is composed of a single player, he is tagged as both player and wall.

#### Multi-view player correspondences and jersey numbers

In the last step, we create one folder per action frame, in which we place that frame along its (possibly multiple) replay frames, and all their corresponding annotations. Each annotator receives a random list of folders to process, thus with frames presumably unseen before. Note that, for this step, the following instructions for players also apply to goalkeepers. Given an action frame and its replay frames, we first ask the annotators to tag the bounding boxes of the players with their jersey number ID, whenever visible. Even if two players on a same frame have the same jersey number, they can still be differentiated by their team ID. Then, we require the annotators to identify which players are visible on multiple images of the folder. In practice, to establish such player correspondences, we ask to propagate the jersey number ID across the instances of a same player when said ID is available for at least one instance of that player. Otherwise, we name the instances of that player with a shared generic literal ID. This annotation strategy for player correspondences is exemplified in Fig. 2 (right). Finally, players appearing only once and without a jersey number ID are not assigned any particular ID. Depending on the targeted application, correspondences between our other annotations may be established without extra annotations for the main referee, the ball, the lines and the goals, when those are visible, given their uniqueness.

## Data Records

The SoccerNet dataset contains the videos for 500 games, split into a training set (300 games), validation set (100) and test set (100). In this work, we release publicly our SoccerNet-v3 annotations for 400 SoccerNet games with a 290/55/55 split. Our training and validation sets do not overlap with the original test set, which contains our test set. This allows for training on one dataset and testing on the other without risking to overfit on any test set. We keep private our SoccerNet-v3 annotations for some of the remaining games of the original test set. They will constitute a challenge set for a future computer vision competition. The statistics presented in this paper are related to the 400 games involved in our public annotations.

Table 1 presents the number of annotations collected at each step, as well as a general overview of all the annotations now available for the SoccerNet games. The SoccerNet and SoccerNet-v2 annotations cover the 500 games of the dataset, excepted for the camera change timestamps spanning 200 games. SoccerNet-v3 annotations of action timestamps within replays cover 400 games, and the remaining annotations are further performed on 33,986 frames (12,764 live action frames and 21,222 replay action frames) spread across those games. They can be accessed through a figshare record^39^ composed of two .zip archives. The first one is named “Full dataset” and contains the frames and their annotation files. The second one is named “Code, Readme, Usage notes” and contains the code described hereafter with a practical guide (README.MD) for installing, downloading, and processing the data. This code is also available on our public Github repository https://github.com/SoccerNet/SoccerNet-v3/.

**Table 1:** SoccerNet panorama.

Version	Annotations	#Instances	#Classes
SoccerNet	Action timestamps + tag of home/away team performing the action	6,637	3
SoccerNet-v2	Action timestamps + home/away tag + shown/unshown tag	110,458	17
	Camera change timestamps and camera used + transition type + “liveness”	158,493	13
	Replay clips delimitation timestamps + link to replayed action	32,932	—
SoccerNet-v3	Total number of frames of interest (action frames + replay frames)	33,986	—
	Action timestamps within replay clips (equals number of replay frames)	21,222	17
	Points placed on field lines (defining 167,589 field lines)	541,411	20
	Points placed on goal parts (defining 53,577 goal parts)	111,286	6
	Bounding boxes for humans (left/right player/goalkeeper, main/side referee, staff)	344,660	7
	Bounding boxes for salient objects (ball, flag, yellow/red card, wall of players)	26,939	5
	Player correspondences between different views of a same scene	172,622	—
	Jersey numbers (including those obtained from player correspondences)	106,592	—

This Table presents a summary of all the annotations related to the SoccerNet video dataset. While the annotations of the previous versions were only composed of timestamps, our 1,324,732 annotations of SoccerNet-v3 are mostly anchored within the spatial domain, allowing for a finer analysis of the scene.

**Fig. 3:**
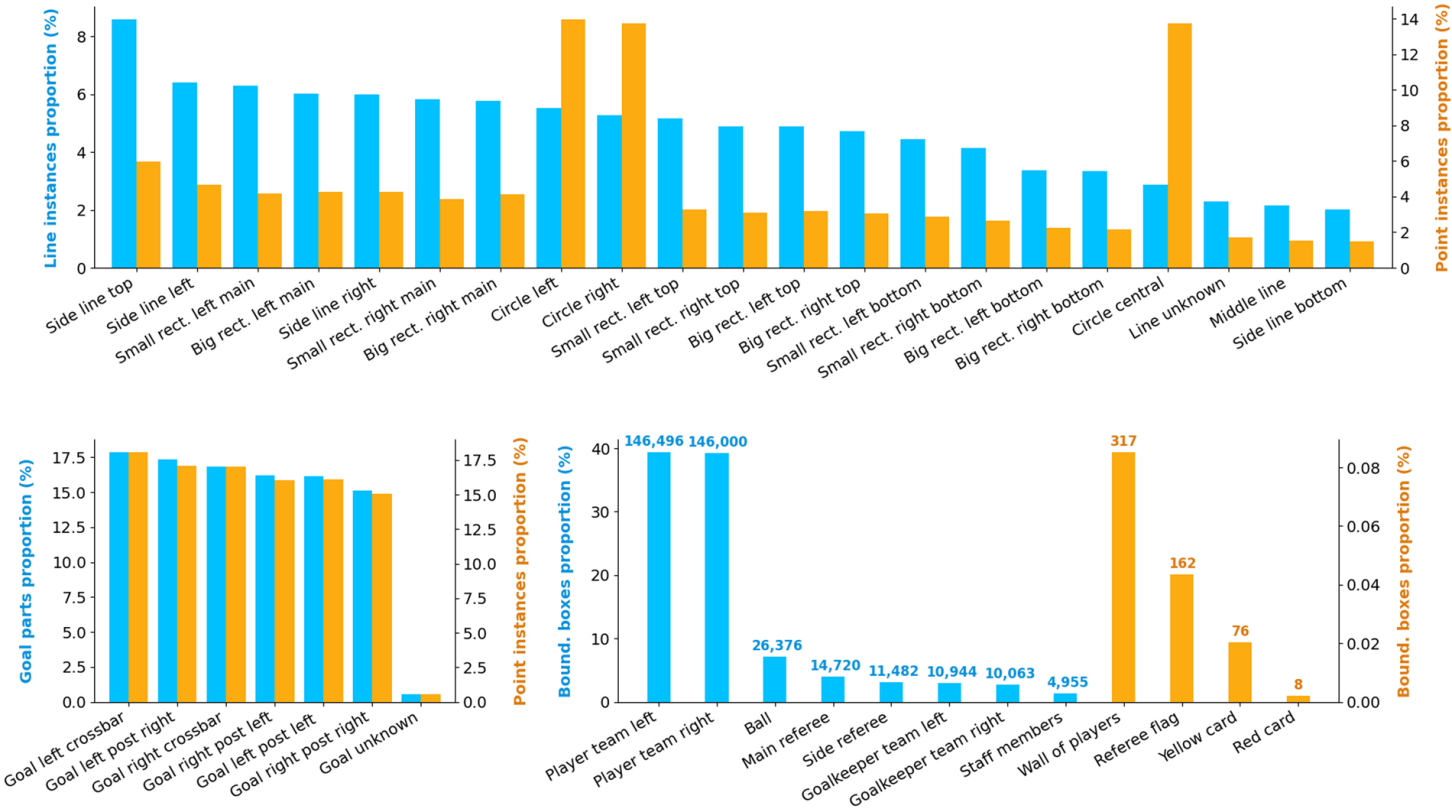
Annotation distributions. Top: distribution of the number of line instances annotated (blue), and of the number of points placed on those lines (orange). Bottom: (left) the same distributions for goal parts; (right) distribution of the bounding boxes annotated, all classes included, with the corresponding number of boxes. A different scale is used for rare objects (orange) for the sake of clarity. Those distributions are computed from our public annotations of 400 SoccerNet games.

Figure 3 shows the distribution of line and goal instances, points placed on lines and goals, and bounding boxes for humans and objects of interest. Regarding line instances, our annotations are relatively well-balanced across the classes, slowly decreasing from 8.6% of instances for the most shown line (side line top) to 2% for the least shown (side line bottom). However, in terms of number of points placed on lines by our annotators, curved lines (circle left, circle right, circle central) account for 41.4% of the total, with the remaining points spread across the other classes following a similar trend as line instances distribution. We can justify this observation by noting that an accurate annotation of a curved line requires many points to properly fit the curvature, while in general it only needs two points for a straight line, except when the camera distortion is too large. Goal instances and points placed on goals do not display such a difference, and are roughly uniformly distributed as well, apart for the few (0.5%) unspecified goal parts. As far as bounding boxes are concerned, 84.4% relate to a player or a goalkeeper and 7.1% to the ball. Rare classes (wall of players, flag, cards) only account for 0.15% of the bounding boxes, which implies that detecting them can be particularly challenging.

## Technical Validation

Two students were hired as proofreaders. Their role was to check the quality of the annotations and alleviate difficult cases. We were in direct contact with them in case of doubt. They were randomly checking the data and correcting potential mistakes. Besides, at each annotation step, the annotators received images randomly. This ensures that the same image was seen and annotated by multiple people, with the possibility to correct previous annotations at each step. Indeed, it is easier to spot someone else’s mistakes than our own, for instance in the case of a recurrent bias such as an annotator never considering a particular class of object. The annotation process was split into 4 consecutive annotation tasks: (1) select the replay clip, (2) annotate the bounding boxes, (3) annotate the lines, and (4) annotate the links between bounding boxes. Once an image was processed for one task, it was automatically sent to the next task, and hence to different annotator, allowing for a fluid pipeline. Hence, each image of interest has been reviewed several times. Over the course of the annotation steps, some images were removed from the pipeline based on annotators feedback to keep only those relevant for our use case. We believe that we did our best to mitigate the risk of label noise. On top of that, we release a data loader and a visualization tool to facilitate the exploration of the dataset and its annotations (see hereafter). This allows users to further check the reliability of our data.

## Usage Notes

Our SoccerNet-v3 dataset, as a self-contained dataset of frames and annotation files, is available at https://www.soccer-net.org/ and will be downloadable freely, with no restriction whatsoever (open licence), as per the fair use principle. For the sake of simplicity, we also provide our 33,986 images of interest directly in the png format, compressed in zip archives. All these ressources can already be downloaded via the SoccerNet pip package. Our annotation files contain the references of the games from which the frames were extracted. The SoccerNet videos of those games, with the initial annotations, and the SoccerNet-v2 annotations can be found on the SoccerNet website, but are not needed to use SoccerNet-v3, as mentioned above. Access to the videos is granted to everyone upon filling out a Non Disclosure Agreement, set up by the authors of the original dataset.

**Fig. 4:**
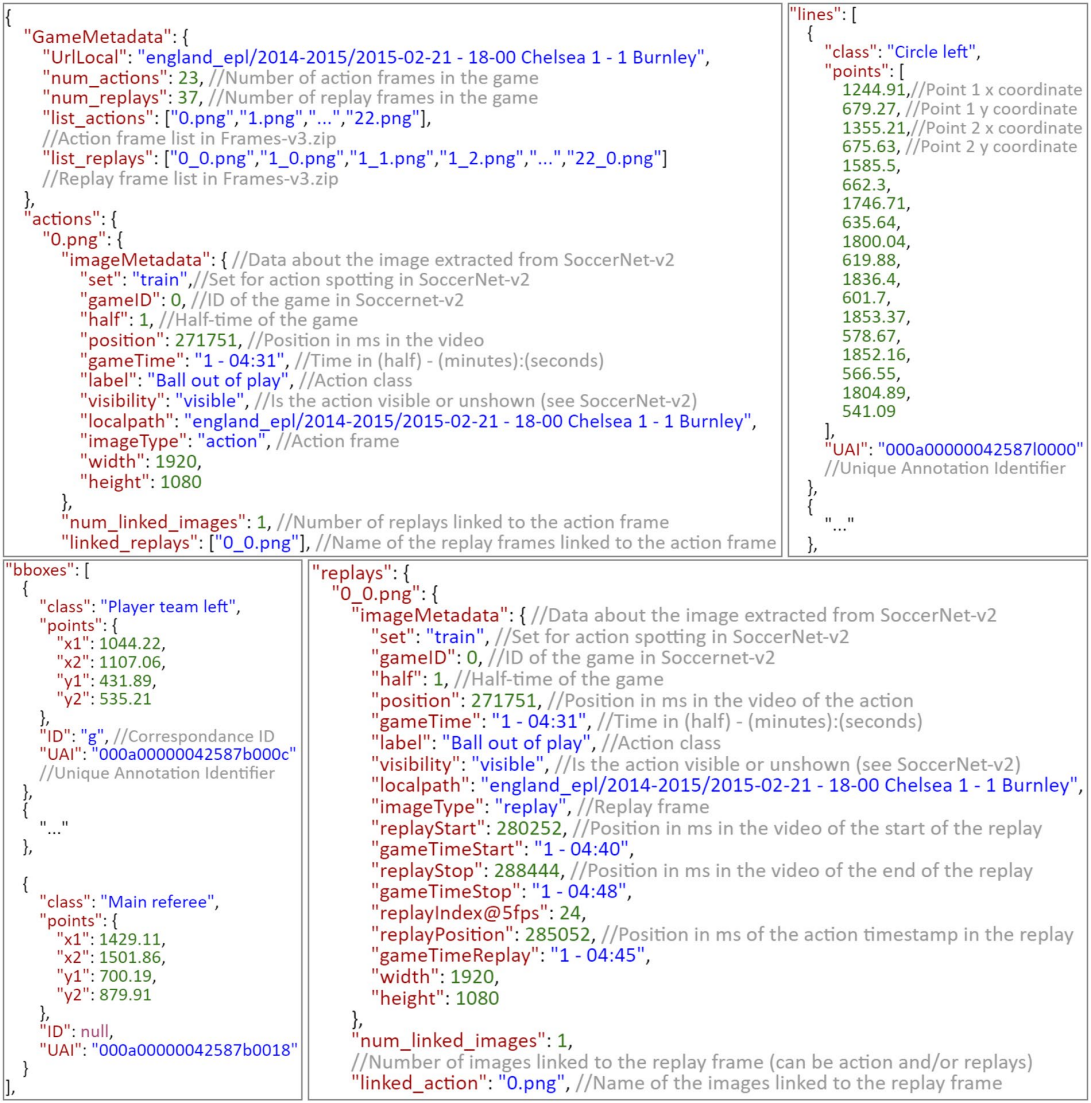
Annotation file format. Our SoccerNet-v3 annotations consist in one.json file per game, as in SoccerNet. Each file contains aggregated information about the annotations within the game (top left). For each action frame annotated, we provide its SoccerNet-v2 information plus its corresponding replay frames (top left), followed by the list of lines annotations (top right), and the list of bounding boxes with player ID (bottom left). Replay frames are treated similarly (bottom right), with information on start time, end time, and localization time of the replayed action appended to the frame metadata. We assign a Unique Annotation Identifier (UAI) to each line or box annotation, to facilitate future data quality assessment and management.

We structure our annotations in json files similarly to SoccerNet, as shown in Fig. 4. For each annotation, we retain the name of the student who performed the annotation for GDPR reasons. This preserves the student’s privacy while being compliant with growing European Union regulatory policies regarding AI and traceability^40^. Also, to ease future data quality assessment and management, we assign a Unique Annotation Identifier (UAI) to each line or box annotation. This allows users to report annotation mistakes efficiently, which we can then correct in future updates of the dataset with a complete traceability.

To facilitate installation and experimentation, we supply a detailed README.MD file on our public github repository https://github.com/SoccerNet/SoccerNet-v3/. This document first explains how to set up an appropriate conda environment and how to download the data. Then, it details how to use three important codes:

1. **dataloader.py** allows to load images from the train, validation and test splits, and the corresponding annotations in the desired resolution and with a generic format for the bounding boxes, lines and correspondences.
2. **visualization.py** allows to draw the annotations on the images. This can be used to check their reliability.
3. **statistics.py** allows to compute the main statistics related to the dataset presented in this paper.

Finally, we review the potential use cases of our annotations. First, camera calibration is the link between the image world and the physical 3D world. Automatic calibration of the camera is an important topic of research for sports analytics that can lead to interesting applications such as offside line analysis, retrieving player statistics such as the estimated distance run, or shot speed estimation. It is also the key to integrate augmented reality graphics into any live production. Some companies have recently started integrating personal advertisements on banners and automatic special effects, which requires a very precise camera calibration. Second, re-identifying soccer players is particularly useful for player tracking across multiple cameras. This will be a key component for building advanced and automatic solutions for highlights generation, such as customized videos focusing on a single player, or for developing better tools to support VAR referees, as well as providing better statistics per player.

## Data Availability

The codes are provided in a figshare^39^ repository and are also available in a public github repository https://github.com/SoccerNet/; the last also provides codes for different baselines for solving tasks, such as camera calibration or re-identification. Those data are used in the public SoccerNet Challenge 2022 for the tasks of camera calibration, pitch localization and player re-identification. Finally, all information about the dataset, the tasks and the challenges can be found on the https://www.soccer-net.org website.
